# Staphylococcal superantigen-like protein 10 enhances the amyloidogenic biofilm formation in *Staphylococcus aureus*

**DOI:** 10.1186/s12866-023-03134-y

**Published:** 2023-12-07

**Authors:** Shakilur Rahman, Amit Kumar Das

**Affiliations:** Department of Biotechnology, Indian Institute of Technology Kharagpur, Kharagpur, 721302 West Bengal India

**Keywords:** Staphylococcal superantigen-like protein, SSL, Biofilm, Amyloid, Aggregation

## Abstract

*Staphylococcus aureus* is a highly infectious pathogen that represents a significant burden on the current healthcare system. Bacterial attachment to medical implants and host tissue, and the establishment of a mature biofilm, play an important role in chronic diseases such as endocarditis, osteomyelitis and wound infections. These biofilms decrease bacterial susceptibility to antibiotics and immune defences, making the infections challenging to treatment. *S. aureus* produces numerous exotoxins that contribute to the pathogenesis of the bacteria. In this study, we have identified a novel function of staphylococcal superantigen-like protein 10 (SSL10) in enhancing the formation of staphylococcal biofilms. Biofilm biomass is significantly increased when SSL10 is added exogenously to bacterial cultures, whereas SSL2 and SSL12 are found to be less active. Exogenously added SSL10 mask the surface charge of the bacterial cells and lowers their zeta potential, leading to the aggregation of the cells. Moreover, the biofilm formation by SSL10 is governed by amyloid aggregation, as evident from spectroscopic and microscopic studies. These findings thereby give the first overview of the SSL-mediated amyloid-based biofilm formation and further drive the future research in identifying potential molecules for developing new antibacterial therapies against *Staphylococcus aureus*.

**Supplementary Information:**

The online version contains supplementary material available at 10.1186/s12866-023-03134-y.

## Introduction

Bacterial biofilm is a complex microbial structure that primarily attaches bacteria to a surface by an extracellular matrix [1]. Since introducing the biofilm model over 45 years ago, researchers have found that most bacteria can form bacterial biofilm as a part of their sustainability. Members of the *Staphylococcus* genus, including *Staphylococcus aureus* and *Staphylococcus epidermis*, can produce rugged multi-cellular biofilms on biotic or abiotic surfaces. *S. aureus* has the ability to form biofilms on medical devices, mostly on heart implants, catheters and prosthetics which made the bacteria most notorious [2, 3]. A recent report showed that staphylococcal biofilm gives rise to life-threatening infectious diseases such as infective endocarditis, defined as a heart infection that can be found on implanted cardiac devices [4]. Biofilm formation offers bacteria to tolerate harsh environments and an advanced defence system against antimicrobial agents, thereby limiting treatment opportunities [5, 6]. Biofilm-related infections are now associated with an elevated level of mortality and morbidity.

Staphylococcal biofilm development is a complex mechanism with three major stages: (i) initial attachment of *S. aureus* cells to biotic or abiotic surfaces, (ii) production of extracellular polymeric substances (EPS) such as polysaccharides, proteins, teichoic acids and extracellular DNA (eDNA), (iii) biofilm structuring and cellular disengagement. Cellular attachment to biotic or abiotic surfaces is the first step of biofilm formation. Binding to biotic surfaces is facilitated by a group of Microbial Surface Components Recognizing Adhesive Matrix Molecules (MSCRAMMs) proteins. Among these MSCRAMMs, fibronectin-binding proteins (FnBPA and FnBPB), clumping factors (ClfA and ClfB), serine-aspartate repeat family proteins (SdrC, SdrD, SdrE) are the key players for the initial attachment to host matrix components. Besides, binding to abiotic surfaces like catheters, implants, polystyrene surfaces and microtiter plates is often mediated by FnBPs, SdrC and biofilm-associated proteins (Bap) facilitated through hydrophobic and electrostatic interactions [7, 8]. Bap proteins can form insoluble amyloid fibrils and protein aggregates [9]. Functional amyloids play important role in staphylococcal biofilm formation [10]. Amyloid assembly is an attractive building block due to its resistance towards harsh denaturing conditions and protease degradation [11, 12].

Staphylococcal superantigen-like proteins (SSLs) are a family of exotoxins composed of 14 members [13]. Though they share structural and sequence similarities with conventional superantigens, they lack mitogenic activities [14, 15]. SSLs possess diverse activities by targeting the host immune proteins. SSL1 interacts with human ERK2 [16], SSL3 binds with toll-like receptor 2 (TLR2) [17], SSL5 targets P-selectin glycoprotein ligand-1 (PSGL-1) [18], SSL7 binds with IgA and complement c5 ^15^, SSL10 interacts with human IgG [19], SSL11 contributes to neutrophil inhibition [20], SSL12 activates mast cells for allergic inflammation [21], SSL13 activates neutrophil via the formyl peptide receptor 2 (FPR2) [22].

Among all the SSL proteins, SSL10 is the most studied staphylococcal toxin reported in several pathological processes. By binding to C-X-C chemokine receptor type 4 (CXCR4), SSL10 inhibits the migration of leukaemia cells [23]. SSL10 blocks the interaction between IgG and complement component C1q, thereby inhibiting the activation of the classical complement pathway [19, 24]. Besides, SSL10 interacts with factor X_a_ and prothrombin, resulting in impaired blood coagulation [25]. All these studies suggest that SSL10 offers multiple functions during *S. aureus* infection, accentuating its importance. In contrast, SSL2 and SSL12 are less-characterized proteins. So, SSLs have been selected to unravel unique functions from the mixture of well-characterized and less-characterized SSLs. In this study, we provide insights into the novel role of SSL10 and propose a molecular mechanism by which SSL10 can promote increased biofilm formation. Using biophysical and microscopic analyses, we report that SSL10 can promote cellular aggregation by lowering the zeta potential and possess amyloid amyloid-like properties, which in turn increases staphylococcal biofilm formation.

## Materials and methods

### Recombinant protein production

Staphylococcal *ssl2* (UniProt: Q2G0 × 8), *ssl10* (UniProt: Q2G2 × 7) and *ssl12* (UniProt: Q2FZB3) *genes* were amplified by polymerase chain reaction using genomic DNA of *Staphylococcus aureus* NCTC 8325 as a template using forward and reverse primer as given (Supplementary Table 1). The amplified ssl2, ssl10 and ssl12 PCR product was purified and cloned into *Bam*HI and *Kpn*I digested pQE30 expression vector (Qiagen, USA). The recombinant plasmid DNA containing ssl2, ssl10 and ssl12 were transformed into chemically competent *E. coli* M15 (pREP4) cells and subsequently selected on ampicillin/kanamycin plates. Positive clones of ssl2, ssl10 and ssl12 were grown in Luria-Bertani broth supplemented with ampicillin (100 µg/mL) and kanamycin (25 µg/mL) at 37 ºC until OD_600_ reached 0.6, followed by induction with IPTG at 15 °C. The SSL2, SSL10 and SSL12 proteins were subjected to size-exclusion chromatography using Superdex 75 prep-grade matrix in a 16/70 C column (GE Healthcare Biosciences) equilibrated with Tris buffer (50 mM Tris–HCl pH 8.0 and 250 mM NaCl).

### Crystal violet assay

*Staphylococcus aureus* NCTC 8325 was grown overnight in 10 mL of Tryptic soya broth (TSB) medium. Primary bacterial culture was diluted to 200-fold in fresh TSB medium, and 200uL (10^6^ CFU/mL) culture was distributed in each well of a 96-well plate (Nunc, Rochester, NY, USA). Increasing concentrations (1µM, 5µM, 10µM and 15µM) of SSL2, SSL10, SSL12, BSA and lysozyme were added exogenously in the wells containing *S. aureus* NCTC 8325 and *S. aureus* MTCC 3160 culture. Plates were incubated at 37 °C for 18 h without shaking for biofilm formation. The culture was discarded, and the wells were washed using 1x PBS 3 times. Crystal violet solution (0.01%) was added to each well and incubated for 15 min, followed by washing another three times using 1x PBS. Ethanol (95%) was added to each well and incubated for 30 min. Finally, the eluted stain was quantified by measuring the absorbance at 595 nm using a BIORAD iMARK microplate absorbance reader (California, United States).

### Cell aggregation assay and surface charge measurement

Cell aggregation and surface charge measurement studies were performed based on a previous report [26]. A 20 mL *S. aureus* inoculation was prepared from overnight culture in tryptic soya broth (TSB) and equally divided into four 50 mL tubes. The first tube contained only bacterial cell culture. Other tubes contained bacterial culture and either 5 µM purified protein or 5 µM BSA, or 5 µM lysozyme, where BSA and lysozyme were used as controls. All the tubes were incubated for 18 h at 37 °C with 160 rpm. Later, tubes were kept for 4 h at 25 °C in static conditions to allow cells to be settled down. Supernatants from each tube were collected without interrupting the aggregation layer. Cell densities were measured at 600 nm for each collected supernatant. Aggregated cell suspensions from each tube were collected and dissolved in PBS. Further bacterial cell surface was measured using DLS Zetasizer (Nano ZS, Malvern Instruments, UK).

### In-silico aggregation analysis

Amyloidogenic regions of protein SSL10 (UniProt: Q2G2 × 7) were determined using phenomenological-based AGGRESCAN [27] and consensus method-based TANGO [28] with their default parameters. AGGRESCAN identifies aggregation ‘Hot spot area’ based on experimentally validated hot-spots. TANGO predicts aggregation-prone areas of a protein based on the physico-chemical principles of β-sheet formation, assuming that the core regions of an aggregate are fully buried.

### Molecular dynamics simulation

Molecular dynamics simulations were performed using the GROMACS 2020.0 package [29] with the OPLS-AA/L force field [30]. Simulation systems were solvated using an SPC water box, and sodium and counter ions were added to neutralize the overall system. A steepest-descent minimization of 5000 steps was performed to release bad contacts in the solvated systems. The system was then slowly heated to 300 K and equilibrated at constant temperature and volume (NVT ensemble), followed by a 100 ns molecular dynamics run at constant temperature (300 K) and the constant pressure of 1 atm (NPT ensemble). All simulations were done with only hydrogen-containing bonds constrained as implemented in GROMACS 2020.0. An integration time step of 2 fs was used, and structures were saved every 10 ps interval for analysis. During NVT and NPT equilibration, the temperature of the system was regulated using the Berendsen thermostat coupling along with a 12 Å short-range electrostatic cut-off. The long-range electrostatic interactions were treated using the particle mesh Ewald (PME) [31] method with a 12 Å cut-off. MD trajectories generated after 100 ns simulation are subjected to various analyses to understand the dynamics of the complexes.

### Preparation of aggregates

The purified protein (1.0 mg/ mL) was incubated at 37 °C for 24 h at 160 rpm.

### Far-UV CD spectroscopy

Far-UV CD spectra of native protein and aggregates were measured on a J-1500 CD spectropolarimeter (JASCO International Co. Ltd., Japan) at a spectra range of 190 to 260 nm.

### Thioflavin T fluorescence assay

Thioflavin T (ThT) is a fluorescent dye that can bind with mature amyloid fibrils. The binding of ThT with fibrils increases the fluorescence intensity. ThT was dissolved in water to prepare a 200 µM stock solution. During the reaction, the final concentration of ThT and protein aggregation was kept at 25 µM and 50 µM, respectively. ThT fluorescence emission spectra were recorded with the spectrofluorometer FluoroMax-4 (HORIBA Scientific, Japan) using the excitation wavelength of 450 nm and emission range of 470 to 600. For both excitation and emission, a 5 nm slit width was used.

### Scanning electron microscopy analysis

*S. aureus* culture was incubated for 18 h in a 6-well culture plate with a sterile 18 mm glass coverslip at the bottom of the well. The culture was added to the wells, followed by the addition of 5 µM protein. After incubation, coverslips were washed with phosphate buffer three times and dried. Coverslips were coated with gold and observed with field emission scanning electron microscopy (FE-SEM; SUPRA™ 40, Carl ZEISS AG, Germany).

### Transmission electron microscopy

Amyloid fibrils were centrifuged at 15,000 x g for 10 min, and 5 µL pellet was applied to a carbon-coated 300 mesh copper grid (Ted Pella, USA) and immediately blotted for excess liquid. Data were acquired using a TECNAI G [2] TF20-ST (FEI, USA) operating at 200 kV.

### Atomic force microscopy

*S. aureus* culture was incubated for 18 h in a 12-well culture plate with a sterile 18 mm glass coverslip at the bottom of the well. The culture was added to the wells, followed by the addition of 5 µM protein. After incubation, coverslips were washed with phosphate buffer three times and dried. Coverslips were observed with atomic force microscopy (AFM; Agilent Technologies, USA).

### Statistical analysis

All experiments were performed in a triplicate base for quantification. The data were represented as mean ± standard deviation. Statistical comparisons were performed using Student’s t-test, where *P* < 0.05 was considered to be statistically significant.

## Results

### Biofilm formation by exogenous addition of SSLs

**Fig. 1:**
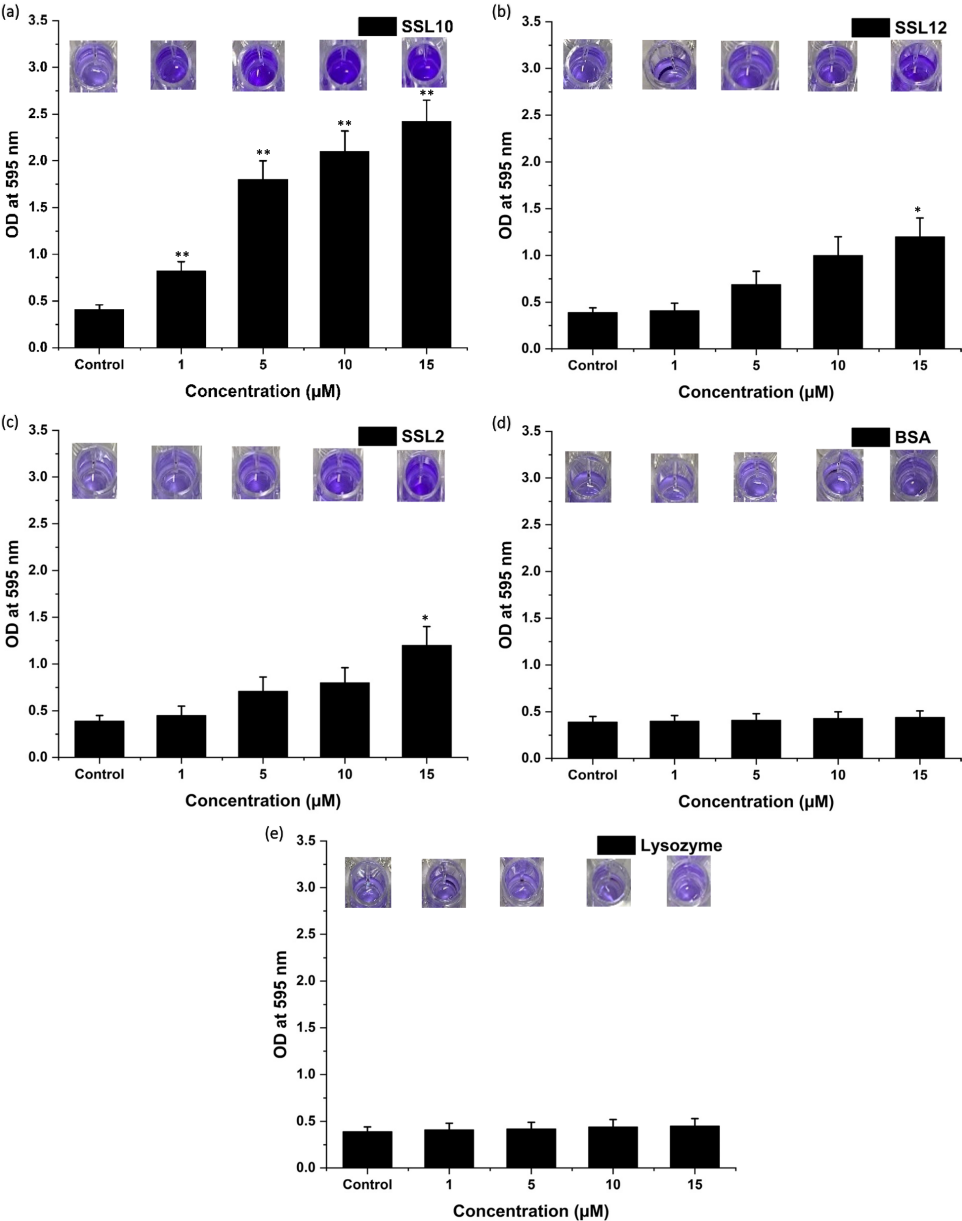
Crystal violet assay to assess increased biofilm formation due to exogenous addition of SSLs, BSA and Lysozyme. (**a**) Significantly increased biofilm after SSL10 treatment, (**b**) SSL12 and (**c**) SSL2 could not significantly increase biofilm formation in the bacterial culture, (**d**) BSA and (**e**) Lysozyme were used as controls that could not induce increased biofilm formation. The statistical significance of data has been represented by *P *< *0.05 and **P *< *0.01

*S. aureus* NCTC 8325 was grown in biofilm conditions with or without SSLs. Crystal violet assay semi-quantitatively assessed the biofilm induction abilities of SSLs in bacterial cultures. Assays showed that the exogenous addition of SSL10 enhanced the biofilm biomass. Upon increasing concentrations of SSL10, the number of bacterial cells that were adhered to the wells of the 96-well plate was also increased. The absorbance of bound crystal violet showed that even adding 1 µM SSL10 can significantly increase biofilm formation by two-fold compared to *S. aureus* control (Fig. 1a). In contrast, when added exogenously to the cultures, SSL12 or SSL2 exerted less activity in biofilm formation (Fig. 1b and c). At the same time, increasing concentrations of lysozyme and BSA failed to enhance the biofilm formation (Fig. 1d and e). CFU assay showed that all SSLs did not increase the growth of the organism. Almost no variation in growth has been observed with respect to the control (3.5 × 10^9^ CFU/mL).

### In-silico identification of amyloidogenic regions in SSL10

**Fig. 2:**
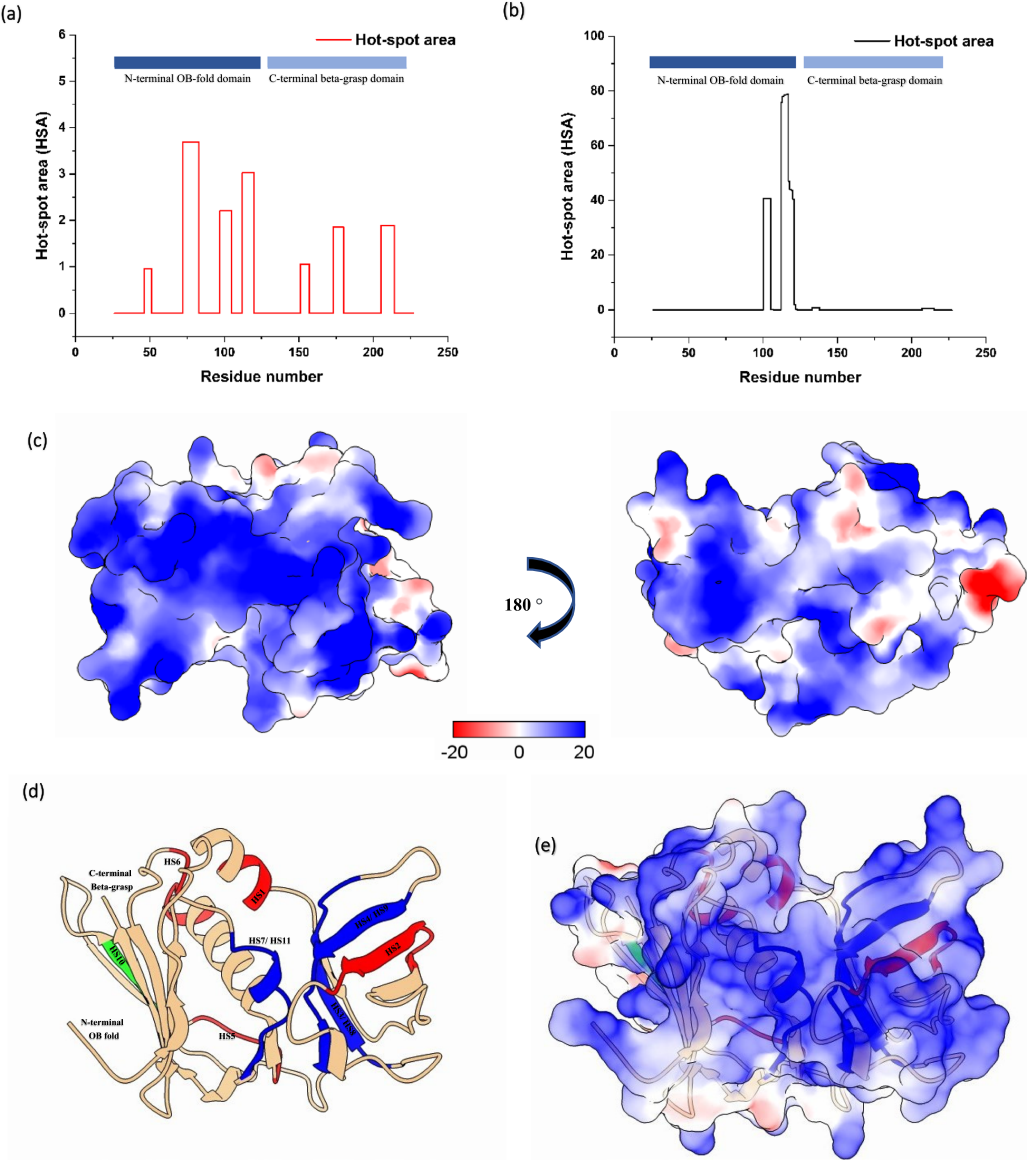
*In-silico* analysis of SSL10 upon amyloidogenic aggregations and electrostatic potential of SSL10. (**a**) Hot-spot are predicted by AGGRESCAN, (**b**) Hot-spot are predicted by TANGO, (**c**) Electrostatic potential of SSL10 shows one side of the protein is abundant with positively charged residues, (**d**) In cartoon representation, red areas indicate the amyloidogenic regions predicted by AGGRESCAN, green areas indicate the amyloidogenic regions predicted by TANGO and blue areas are the common ‘hot-spots’ predicted by both the tools (**e**) Amyloidogenic regions of N-terminal OB-fold are mostly lying with the positively charged surface. Visualization of electrostatic potential was executed using the UCSF ChimeraX 1.5

 Aggregation-prone regions (APRs) of staphylococcal superantigen-like protein-10 were primarily determined by the presence of amyloidogenic regions in its structure. Based on the algorithms embedded in both AGGRESCAN and TANGO tools, aggregation-prone “hot spot area” (HSA) in SSL10 (Fig. 2a and b; Table 1) was predicted. These hot-spot regions are depicted in Fig. 2d, where red regions were predicted by AGGRESCAN, the green region was predicted by TANGO and blue regions were predicted by both tools. AGGRESCAN predicted HS1 is present in the α1-helix, HS2 region lies in the first β turn, HS3 is present in the β4-sheet, HS4 is found to be in the β6-sheet, HS5 is present in the loop between β8-sheet and β9-sheet, HS6 lies at the end of α2 helix, and HS7 is present in the α3-helix. Besides, TANGO-predicted HS8 is found to be in the β4-sheet which is common to AGGRESCAN-predicted HS3, and HS9 lies in the β6-sheet, which is common to AGGRESCAN-predicted HS4. HS10 is the only uncommon between the two tools and is present in the β7-sheet. HS11 is found to be shared with HS7 and extended up to the β12-sheet. Furthermore, the area of the first four “hot spots” predicted by AGGRESCAN (located within the OB domain 44–123) is larger than the areas of the C-terminal β-grasp domain (133–226). Three HSAs (100–106, 112–121, 207–214) are common in both the tools and found to be present in the OB and β-grasp domain of SSL10, which might contribute to aggregation. The distribution of electrostatic potential was evaluated based on the available crystal structure of SSL10 (PDB: 6UCD). Interestingly, the electrostatic map of SSL10 showed an asymmetric distribution of positive and negative charges on the protein surface. One side of the protein possesses highly positive charge regions, whereas the other side of the protein contains positive, neutral and negatively charged regions (Fig. 2c). Overall, the positive charges are abundant on the SSL10 surface (having a net charge of + 20). Amyloidogenic regions HS1, HS2, HS3, HS5, HS6, HS7 (highly positive), HS8 and HS11 (highly positive) are mostly present in positively charged regions, whereas HS4, HS9, HS10 lie in a neutral area (Fig. 2e).

**Table 1:** Amyloidogenic ‘hot-spot’ regions predicted by AGGRESCAN and TANGO. Common regions are denoted with blue

AGGRESCAN	Amyloidogenic regions	Sequence range	Region	Secondary structure element
	YRYYT	46–50	HS1	α1-helix
	KFRGIKIQVLL	72–82	HS2	First β-turn
	GLDVFFVQ	97–104	HS3	β4-sheet
	IFYTVGGV	112–119	HS4	β6-sheet
	YYIKKE	151–156	HS5	Loop between β8-sheet and β9-sheet
	EKYGLYK	173–179	HS6	end of α2-helix
	LKFKYMGEV	205–213	HS7	α 3-helix
**TANGO**	VFFVQEK	100–106	HS8	β4sheet
	IFYTVGGVIQ	112–121	HS9	β6-sheet
	ILNIS	133–137	HS10	β7-sheet
	FKYMGEVI	207–214	HS11	α3-helix and extended up to β12-sheet

### Essential dynamics, change in secondary structure and free energy landscape of SSL10

**Fig. 3:**
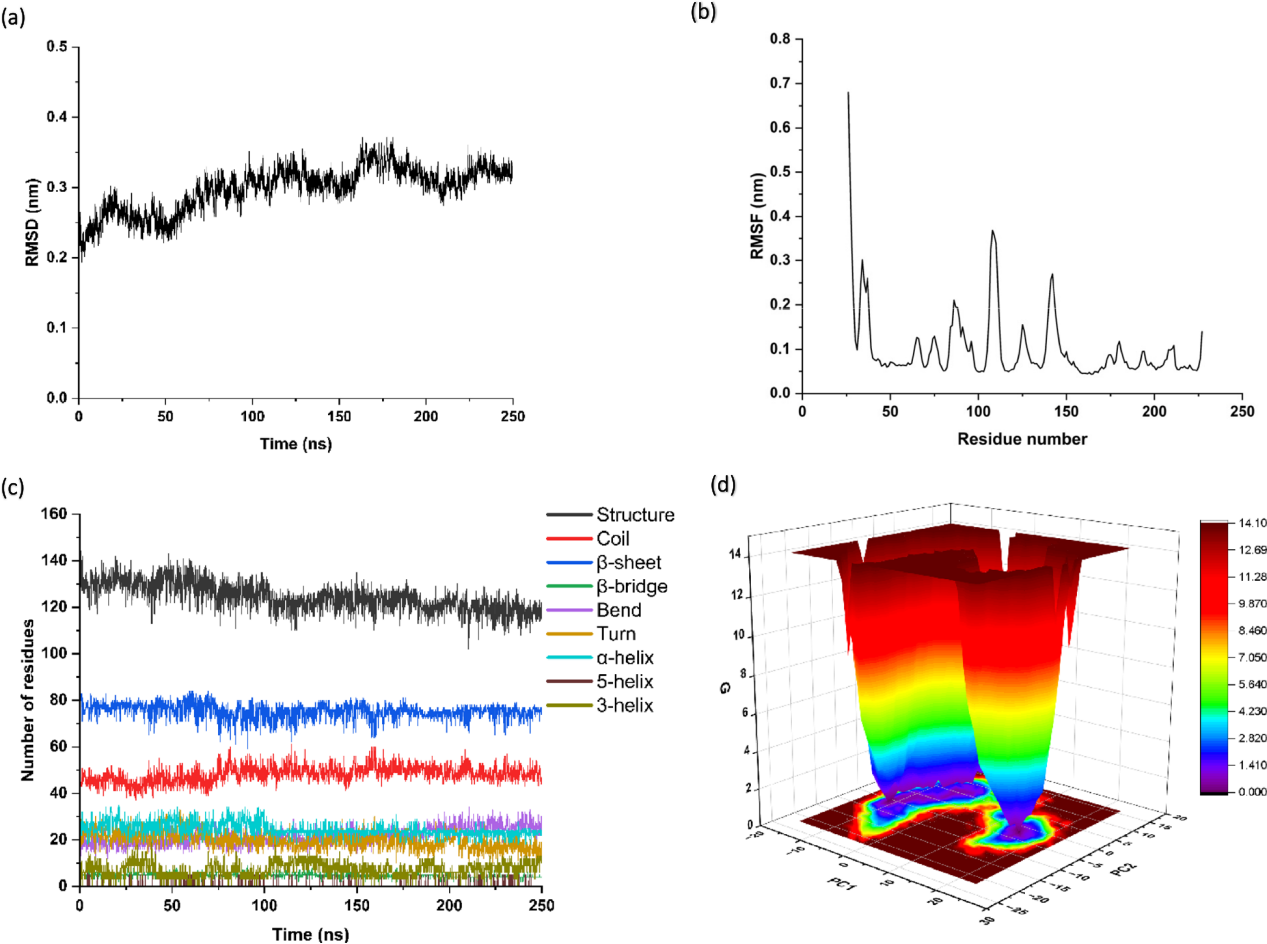
MD simulation analysis of SSL10 to assess structural stability, residual fluctuations, change in secondary structure and free energy landscape. (**a**) Root-mean-square deviation of SSL10 indicating unstability of protein over time, (**b**) Root-mean-square fluctuations of SSL10 residues indicating higher fluctuating regions of SSL10, (**c**) Decreased α-helix residues and increased coil and β-bend indicates the change in SSL10 secondary structure, (**d**) Free energy landscape indicated overall thermodynamic unstability in 250ns MD simulation

 The structural stability of SSL10 in terms of root-mean-square deviation (RMSD) showed that the protein became unstable over the time of the simulation (Fig. 3a). Root-mean-square fluctuation (RMSF) plot suggested that regions 84–92, 106–112 and 139–146 of SSL10 possess higher fluctuations and thus contribute to the unstability of the protein (Fig. 3b). The DSSP algorithm of GROMACS 2020.0 analyzed the overall change in secondary structure components with time. SSL10 showed a considerable decrease in α-helix forming residues with the related increase in β-bends and coils (Fig. 3c). This information indicated the transition of α-helix to β-bends and coils, although increased β-sheet could not be observed with the 250ns simulation. Gibbs free energy landscape (FEL) represents the thermodynamic stability of a given system. FEL of SSL10 was calculated with the inbuilt scripts of GROMACS 2020.0 and the first two principal components (PC1 and PC2) of the system. It is the multidimensional energy map of a system where each dimension represents a particular structural degree of freedom. Multiple minimum energy basin defines the thermodynamic unstability of protein, whereas a single conformation basin indicates a stable structural conformation. In this 3D contour plot of FEL, the dark violet/blue region correspond to the energy minima indicating the thermodynamically favoured conformation, whereas the red/yellow regions represent the opposite. Here, it was observed that the SSL10 structure achieved one distinct basin and a wide, stretched local minima region that indicated the thermodynamic imbalance of the protein (Fig. 3d).

### Cellular aggregation and lowered surface charge

**Fig. 4:**
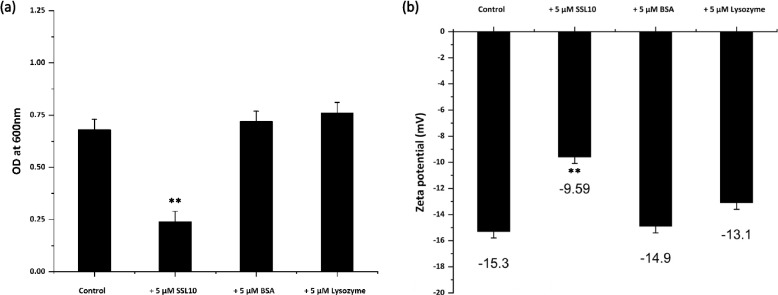
Effect of SSL10 on cellular aggregation and cell surface charge. (**a**) The cell density of *S. aureus* NCTC 8325 cultures in the absence and presence of SSL10, (**b**) Surface charge of *S. aureus* NCTC 8325 measured in the absence and presence of SSL10. The statistical significance of data has been represented by **P *< *0.01

 The role of SSL10 in staphylococcal cell aggregation was further evaluated by aggregation assay. Incubation of the cells with SSL10 caused aggregation of the cells where cells were settled down at the bottom of the tubes. In contrast, the supernatant remained turbid in the control culture, where 5 µM BSA and 5 µM lysozyme were separately added. According to the comparative analysis of the OD_600_ values of the supernatants, the cell density in the supernatant treated with SSL10 was reduced (Fig. 4a). This finding implied that SSL10 plays a part in the aggregation of bacterial cells. Zeta potential measured using DLS indicated the change in surface charge of the cells in the presence of SSL10. The cells, which were incubated with SSL10, showed a lesser negative charge (− 9.59 mV) than those incubated with lysozyme (− 13.1 mV) or BSA (− 14.9 mV). Untreated *S. aureus* NCTC 8325 cells (− 15.3 mV) possessed higher zeta potential in comparison with SSL10-treated cells (Fig. 4b). The binding of SSL10 to the *S. aureus* surface caused masking of the surface charge, leading to lower zeta potential than the control cells. BSA (pI: 4.7) and lysozyme (pI: 11.35) were taken as controls based on the surface charge to observe their effect on bacterial surface charge (Fig. 4b). The lower zeta potential of the cell surface in the presence of SSL10 ultimately resulted in stable precipitation. This precipitation might encourage increased biofilm formation, as shown in the crystal violet assay.

### SSL10 forms amyloid aggregates

**Fig. 5:**
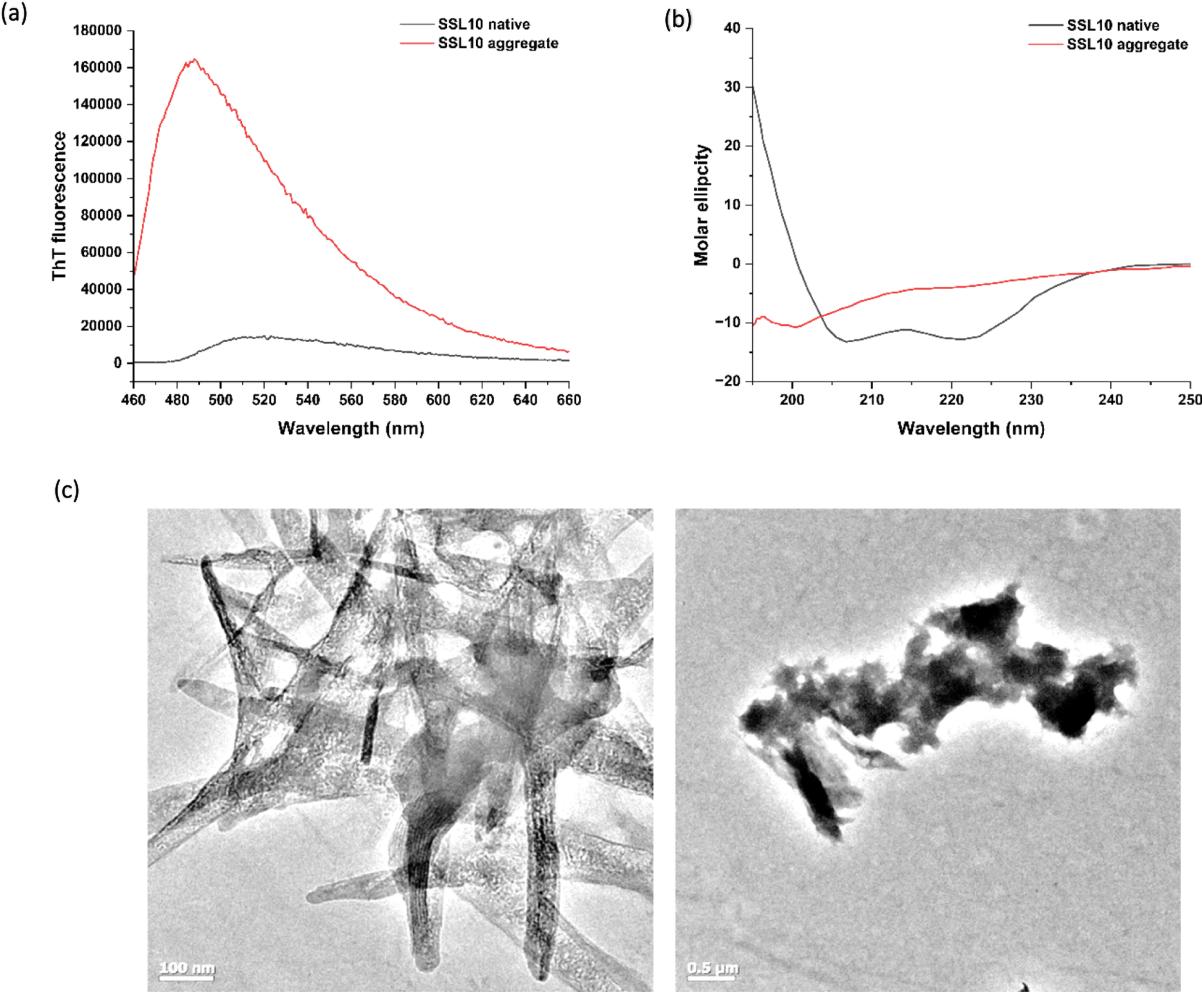
SSL10 forms amyloid aggregates. (**a**) Thioflavin T fluorescence emission spectra of SSL10 in native and aggregation form, (**b**) Circular dichroism spectrum of SSL10 in native and aggregation form, (**c**) Transmission electron microscopy images of SSL10 amyloids

Functional amyloids are important building blocks for bacterial biofilms. Amyloids were assessed using Thioflavin T to investigate aggregations. Protein aggregates were formed and tested with the dyes. Thioflavin T fluorescence assay indicated the presence of high β-sheet structures in the aggregates. Upon binding of ThT with the SSL10, a visible increase in the absorbance was observed in comparison with the control (Fig. 5a). An increase in fluorescence intensity was noticed around 490 nm. ThT also suggested the amyloid aggregation formation by SSL10. An increase in β-sheet resulted in the formation of amyloid aggregations. Circular dichroism spectroscopy measured the secondary changes of SSL10 aggregates. CD spectrums of SSL10 were taken just after purification and after 1 day. The negative minima at 210 nm in the fresh protein indicated the presence of helical conformations (Fig. 5b). Freshly prepared SSL10 protein possessed 47.9% regular α-helix, 23.4% distorted α-helix and 28.6 parallel β-sheet with 0% turn. Besides, aggregated SSL10 sample showed 22.8% α-helix, 65.7% anti-parallel and 11.5% turn. Thus, CD spectra of the aggregation sample reported the change in the secondary structure just after 1 day. Loss of helical structure and gain of β-sheet and turn was observed from the CD spectra. Results from the ThT assay also corroborated the presence of high β-sheet structures in the aggregates. Further transmission electron microscopy (TEM) images confirmed the presence of amyloid-aggregates formed by SSL10 itself (Fig. 5c). Microscopy images showed the presence of multiple type amyloid aggregations of SSL10 (Supplementary Fig. 3).

### Typical biofilm morphology

**Fig. 6:**
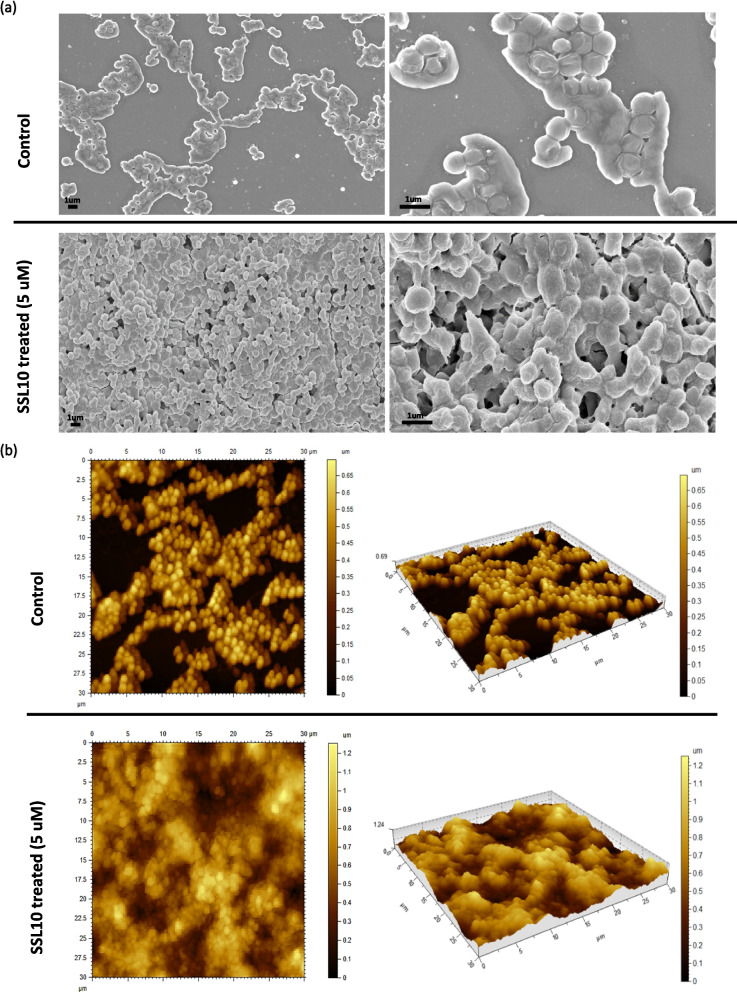
Visualization of increased biofilm biomass in culture due to exogenous addition of SSL10. (**a**) Scanning electron microscopy images of *S. aureus *biofilms where images were taken at x10,000 and x25,000 magnification, (**b**) Atomic force microscopy images of *S. aureus *biofilms

 Staphylococcal biofilm morphology was characterized by scanning electron microscopy (SEM) and atomic force microscopy (AFM). SEM images showed significantly increased biomass growth in treated samples compared to control samples. In the control samples, the number of cells was significantly less, and the cells were observed in a scattered manner (Fig. 6a). *S. aureus* NCTC 8325 formed a thick biomass made of cell aggregates to generate the 3-dimensional biofilm structure. Biofilms grown on cover slides had an enormous amount of densely stacked bacterial cells representing a typical biofilm structure. The SEM micrographs showed the presence of layered biofilm produced by *S. aureus*. Bacteria gave rise to a lump-like structure where cells were bridged with each other with the help of amyloid aggregations (Fig. 6a). Atomic force microscopy provided physical information on the surface of the biofilm. AFM analyses showed the characteristic cocci feature of *S. aureus* NCTC 8325. Increased biofilm production was observed after incubation with SSL10 (Fig. 6b). The largely distributed bacterial population gave rise to the typical biofilm structure. 3D images rendered from AFM demonstrated the presence of cellular aggregates in terms of residual biomass that gave rise to the bacterial biofilm (Fig. 6b). AFM analyses also provided information about surface morphology. Based on the obtained images, the roughness of the biofilm surface was investigated for control and treated samples. Roughness in terms of root mean square height in the treated sample (0.205 μm) was relatively higher compared to the control (0.184 μm). The control sample showed a significantly lower number of cell aggregates in the matrix and flat regions between them. Under the influence of SSL10, the maximum height of biofilm biomass in the treated sample was observed with a value of 1.25 μm compared to the control sample (0.69 μm). These observations supported the topographic difference in biofilm formation in treated and untreated samples.

## Discussion

Biofilm formation is one of the strategies of bacteria to build social groups by which the bacteria are glued together through a matrix; thus, an immobile microbial community colonizes on medical devices or implants. The matrix, an extracellular polymeric substance, is composed of proteins, complex carbohydrates and extracellular DNA. The matrix formed by the bacteria protects themselves from the environment or any stress like an antibiotic. The impact of biofilm formation depends on the type of bacteria that live in the host, causing harm to the host. Thus, the pathogens use the sticky and protective nature of the biofilm matrix, which plays a vital role in infection. *Staphylococcus aureus* is one of such pathogens which causes nosocomial diseases. So, it is very essential to understand the mechanism of biofilm formation and the agents that cause enhanced biofilm formation. In this work, an attempt has been made to understand the effect of a particular class of proteins on biofilm formation caused by the bacteria. Overall, *S. aureus* biofilm formation and drug resistance are major challenges for the treatment of infections caused by this bacterium.

Amyloids have long been linked to a number of incurable degenerative human disorders. Amyloids have recently been recognised as key biofilm components, giving uniformity and viscoelasticity to the extracellular biofilm matrix [32–35]. Amyloids are highly organized fibrillar proteins with a β-sheet secondary structure and a highly conserved quaternary cross-structure. Amyloids are an excellent extracellular building material due to their well-ordered structure of β-strands oriented perpendicular to a fibril axis. They are often resistant to denaturing agents and proteolytic cleavage. Furthermore, in the absence of energy, polymerization of amyloidogenic proteins happens via a nucleation-dependent self-assembly process in which starting amyloid aggregates provide a conformational framework that enables the assembly of polymeric subunits into the amyloid state. Because of this seeding process, the amyloid structure is appropriate in settings when energy is restricted [32].

*S. aureus* releases varieties of superantigens (SAgs) and superantigen-like proteins (SSLs) that cause immunostimulatory effects. SSLs share the structural similarity with SAg but no binding with MHC II or T cell receptors to evoke a toxic cytokine response. In this study, *ssl2, ssl10* and *ssl12* genes have been cloned into *E. coli* for the production of corresponding recombinant proteins to see their effects on biofilm formation. The biofilm biomass is visualized using SEM, TEM and AFM and biofilm formation is also tested biochemically using crystal violet assay. The effect of exogenously added SSL2, SSL10 and SSL12 protein in the growth of *S. aureus* has been evaluated at 37 °C. It is found that SSL10 can significantly increase the staphylococcal biofilm biomass compared to SSL2 and SSL12. Thus, SSL2 and SSL12 elicit less biofilm induction activity in *S. aureus* culture. CD spectrum indicates the change in the secondary structure of SSL10 at 37 °C, specifically gain in anti-parallel β-sheet has been observed. A transition from α-helix to β-strand facilitates the formation of amyloid fibrils [36, 37]. Thus CD analysis also sheds light on amyloid formation due to the shift of α-helix towards β-sheet. The current study reports that biofilm formation is induced mostly by amyloid fibrils. The Thioflavin T assay also confirms the presence of amyloid fibrils in the biofilm. These fibrils are also visualized by TEM micrographs. Analysis of SSL10 sequences shows the presence of positively charged stretches, which may be involved in protein aggregation via amyloid fibril formation (Table 1; Fig. 2d). SSL10 structure. Molecular dynamics analysis shows the higher fluctuation of these stretches, change in secondary structure and unfavoured thermodynamic conformation, indicating the overall unstability of SSL10, which may lead to the formation of amyloids. MD simulation shows a decrease in α-helix and an increase in β-turn and loop content which also corroborates with the CD spectrum results.

Zeta potential, a measure of the effective electric charge, indicates that the surface charge of the *S. aureus* cell is reduced by the exogenous addition of SSL10, resulting in cellular aggregation and biofilm formation. Larger positive patches on protein show a tendency towards protein aggregation [38]. Net charge calculation shows that recombinant SSL10 has a higher positive charge (+ 20) compared to recombinant SSL2 (+ 3) and SSL12 (+ 6), which corroborates with less biofilm formation by SSL2 and SSL12. AGGRESCAN and TANGO predicted the aggregation-prone hot-spot regions in the SSL10, and these regions play a crucial role in SSL10-mediated amyloid aggregation. The electrostatic potential map of SSL10 shows the abundance of positive charges in the protein surface, and the accumulation of those positive charges on the negatively charged cell surface lowers down the membrane potential causing cellular aggregation. The transition from α-helix to β-sheet and loops are the signature phenomenon of amyloid formation. In this study, DSSP analysis shows a significant change in protein secondary structure where α-helix residues are decreased, and β-bends and coils are found to be increased. Although, a 250ns MD simulation could not provide any information on the α-helix to β-sheet shift. SEM and AFM images show exogenous addition of SSL10 in *S. aureus* culture results in increased biomass production that eventually gives rise to the typical biofilm structure. Overall, this study shows that SSL10 undergoes amyloid-like aggregates and attaches to the cell surface, facilitating the biofilm formation of *S. aureus* NCTC 8325 culture.

Functional amyloids play a crucial role as bacterial biofilm-building elements [10]. These biofilms can facilitate the development and spread of antibiotic resistance. The extracellular matrix of the biofilm can act as a barrier that protects bacteria from antibiotics, promoting the emergence of resistant strains within the biofilm. Therefore, biofilm-forming bacteria like *S. aureus* are often associated with chronic infections that are difficult to eradicate. Overall, *S. aureus* biofilm formation and drug resistance are major challenges for the treatment of infections caused by this bacterium. Effective strategies to prevent and treat biofilm-associated infections will require a better understanding of the underlying mechanisms of biofilm formation and antibiotic resistance, as well as the development of novel antimicrobial therapies.

## Conclusion

*Staphylococcus aureus* biofilm-associated infections are the leading cause of mortality and morbidity. Effective strategies to prevent and treat biofilm-associated infections will require a better understanding of the underlying mechanisms of biofilm formation and antibiotic resistance, as well as the development of novel antimicrobial therapies. SSL10 binds to the bacterial cell, which lowers the membrane potential, followed by cellular aggregation. Thus, SSL10 has the potential to form amyloid structures that help in escalated biofilm formation. This study confirms the role of SSL10 as a biofilm enhancer through amyloid fibril formation. These findings provide the first overview of SSL-mediated amyloid-based biofilm development, which pave the path for further study into the identification of promising compounds against the SSLs for developing novel antibacterial treatments.

### Supplementary Information

**Additional file 1: Supplementary Table 1.** Primers used for PCR amplifications of *ssl2, ssl10, ssl12 *constructs. **Supplementary Figure 1.** 12% SDS-PAGE showing Size-exclusion purification of SSL2. Lane 1: MW Marker, Lane 2: Protein after Gel filtration. **Supplementary Figure 2.** 12% SDS-PAGE showing Size-exclusion purification of SSL10 and SSL12. Lane 1: SSL10 after Gel filtration, Lane 2: MW Marker, Lane 2: SSL12 after Gel filtration. **Supplementary Figure 3.** Multiple types of SSL10 amyloid aggregations were noticed in TEM images.

## Data Availability

All data are given in the manuscript.
