# Supplementary material for: Staphylococcal superantigen-like protein 10 enhances the amyloidogenic biofilm formation in Staphylococcus aureus
Source: BMC Microbiol. 2023 Dec 7;23:390. doi: 10.1186/s12866-023-03134-y (PMC10701973; doi:10.1186/s12866-023-03134-y)
Supplement: Supplementary file 1 — Additional file 1: Supplementary Table 1. Primers used for PCR amplifications of ssl2, ssl10, ssl12 constructs. Supplementary Figure 1. 12% SDS-PAGE showing Size-exclusion purification of SSL2. Lane 1: MW Marker, Lane 2: Protein after Gel filtration. Supplementary Figure 2. 12% SDS-PAGE showing Size-exclusion purification of SSL10 and SSL12. Lane 1: SSL10 after Gel filtration, Lane 2: MW Marker, Lane 2: SSL12 after Gel filtration. Supplementary Figure 3. Multiple types of SSL10 amyloid aggregations were noticed in TEM images. [file 12866_2023_3134_MOESM1_ESM.docx]

**Staphylococcal superantigen-like protein 10 enhances the amyloidogenic biofilm formation in *Staphylococcus aureus***

Shakilur Rahman and Amit Kumar Das *

Department of Biotechnology, Indian Institute of Technology Kharagpur, Kharagpur 721302, West Bengal, India

* Correspondence: amitk@bt.iitkgp.ac.in; Tel: +91-3222-283756

**Supplementary Table 1. Primers used for PCR amplifications of *ssl2, ssl10, ssl12* constructs.**

| **Toxin** | **Forward Primer with Restriction Endonuclease** | **Reverse Primer with Restriction Endonuclease** |
| --- | --- | --- |
| SSL2 | 5’-CG**GGATCC**GAAAAACCAGTTCATG-3’; *Bam*HI | 5’-CGG**GGTACC**TTATGCTTTTATAACTTTGATTTC -3’; *Kpn*I |
| SSL10 | 5’-CG**GGATCC**CATTCAGGTCATG-3’; *Bam*HI | 5’-CCC**AAGCTT**TTACTTTAAGTTAACTTCAATATC-3’; *Hin*dIII |
| SSL12 | 5’-CGC**GGATCC**CAAAAACCAGTATTTAGTTTTTACTC-3’; *Bam*HI | 5’-CGG**GGTACC**CTAATCAAAGAGGACGTCAACAGT-3’; *Kpn*I |

**MW**

**1**

**2**


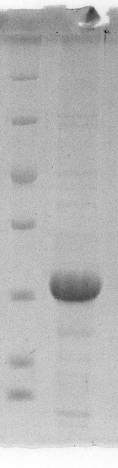


**14.4**

**18.4**

**25**

**45**

**66.2**

**166**

**35**

**Supplementary Figure 1. 12% SDS-PAGE showing Size-exclusion purification of SSL2. Lane 1: MW Marker, Lane 2: Protein after Gel filtration.**

**Supplementary Figure 2. 12% SDS-PAGE showing Size-exclusion purification of SSL10 and SSL12. Lane 1: SSL10 after Gel filtration, Lane 2: MW Marker, Lane 2: SSL12 after Gel filtration.**


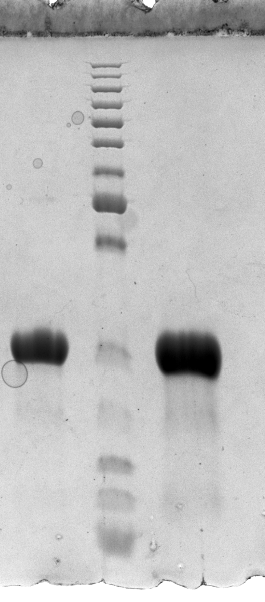


**MW**

**1**

**2**

**3**

**10**

**15**

**20**

**25**

**30**

**40**

**50**

**60**

**70**

**85**

**100**

**120**

**150**

**200**


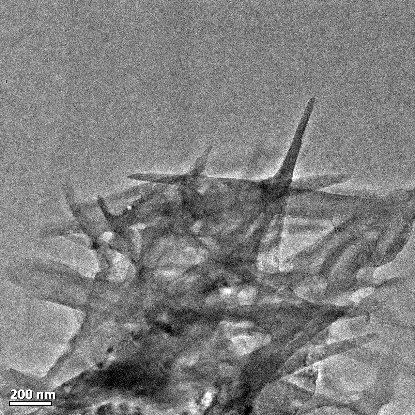

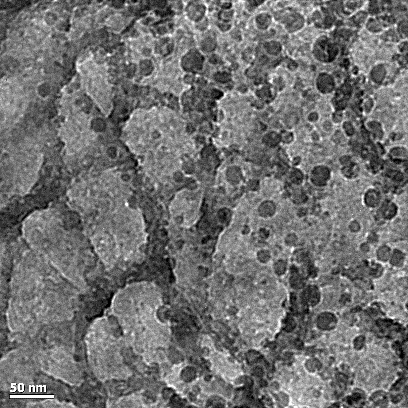

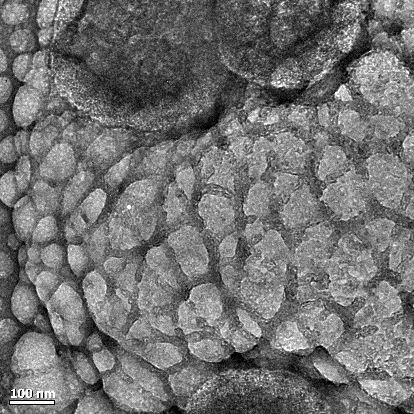


**Supplementary Figure 3. Multiple types of SSL10 amyloid aggregations were noticed in TEM images.**
